# Accurate Rapid Lifetime Determination on Time-Gated FLIM Microscopy with Optical Sectioning

**DOI:** 10.1155/2018/1371386

**Published:** 2018-01-16

**Authors:** Susana F. Silva, José Paulo Domingues, António Miguel Morgado

**Affiliations:** ^1^Institute for Biomedical Imaging and Life Sciences (IBILI), Faculty of Medicine, University of Coimbra, Coimbra, Portugal; ^2^Department of Physics, Faculty of Sciences and Technology, University of Coimbra, Coimbra, Portugal

## Abstract

Time-gated fluorescence lifetime imaging microscopy (FLIM) is a powerful technique to assess the biochemistry of cells and tissues. When applied to living thick samples, it is hampered by the lack of optical sectioning and the need of acquiring many images for an accurate measurement of fluorescence lifetimes. Here, we report on the use of processing techniques to overcome these limitations, minimizing the acquisition time, while providing optical sectioning. We evaluated the application of the HiLo and the rapid lifetime determination (RLD) techniques for accurate measurement of fluorescence lifetimes with optical sectioning. HiLo provides optical sectioning by combining the high-frequency content from a standard image, obtained with uniform illumination, with the low-frequency content of a second image, acquired using structured illumination. Our results show that HiLo produces optical sectioning on thick samples without degrading the accuracy of the measured lifetimes. We also show that instrument response function (IRF) deconvolution can be applied with the RLD technique on HiLo images, improving greatly the accuracy of the measured lifetimes. These results open the possibility of using the RLD technique with pulsed diode laser sources to determine accurately fluorescence lifetimes in the subnanosecond range on thick multilayer samples, providing that offline processing is allowed.

## 1. Introduction

Fluorescence lifetime imaging microscopy (FLIM) can assess the biochemistry of living cells, discriminate between different fluorescent molecules, and provide information on the local environment on the fluorophore [1–3]. When imaging living cells, it is important to obtain the fluorescence lifetime images as fast as possible. This requirement is best suited by wide-field imaging techniques, where image pixels are acquired in parallel, than by scanning techniques with serial pixel acquisition, like time-correlated single photon counting (TCSPC), the most common time-domain FLIM technique [4]. Time-gated FLIM is a wide-field technique where the fluorescence decay is sampled by a time-gated camera, at several delays after the laser pulse used to excite fluorescence [5, 6]. This results in a series of images with decreasing fluorescence intensity. The values of the equivalent pixels of the image volume correspond to multiple samples of the decay curve that can be fitted by least-squares methods for yielding the decay parameters. However, this approach requires the acquisition of many images, which can be unsuitable for *in vivo* applications with low fluorescence signals that require many excitation cycles for each gate delay.

The rapid lifetime determination (RLD) method is a set of algorithms that, instead of recording multiple images for obtaining a complete multipoint decay curve, uses areas under regions of the decay curve to determine the decay parameters [7, 8]. This way, the fluorescence lifetime *τ* associated to a single-exponential decay *F*(*t*, *τ*) can be determined using just two images (*I*_0_ and *I*_1_), corresponding to two acquisition windows (usually called gates) of equal width Δ*t*, set at distinct delays.

(1) $$\begin{array}{c} F\left(t,\tau \right)=F_{0}\exp\left(-\frac{t}{\tau }\right),\\ \tau =\frac{-∆t}{\ln I_{1}/I_{0}}. \end{array}$$

This approach to FLIM presents limitations. On one hand, it is necessary to have a priori knowledge of the expected lifetimes to maximize the signal-to-noise ratio and, in the presence of a mixture of fluorophores with different lifetimes, the RLD algorithm only outputs an effective lifetime value that corresponds to an average lifetime weighted by the fluorophore's relative contribution. Moreover, time-gated FLIM is a wide-field imaging technique that does not provide optical sectioning. Finally, (1) is only accurate if the excitation laser pulse width is at least two orders of magnitude shorter than the fluorescence lifetime. This last requirement implies the use of expensive femtosecond lasers when measuring subnanosecond fluorescence lifetimes.

Here, we report on the use of processing techniques to overcome the limitations arising from the lack of optical sectioning and from the finite width of the laser pulse. Our goal is to minimize the number of images required to retrieve accurately the fluorescence lifetime, while providing optical sectioning. This is an important goal when imaging living tissues.

## 2. Time-Gated Fluorescence Lifetime Microscope

We assembled a time-gated fluorescence lifetime microscope. The light source of the microscope is based on a pulsed diode laser (LDH-P-C-440M, PicoQuant, Berlin, Germany), with a central wavelength of 440 nm and a pulse rate up to 40 MHz, controlled by a multichannel picosecond laser driver (PDL 828 “Sepia II”). The laser output has a maximum average power of 22.5 mW. Peak power is adjustable by the user. This adjustment affects the pulse width that can vary between 63 and 190 ps (FWHM).

The time-gated acquisition system is based on a gated-intensified camera (PicoStar HR, LaVision, Goettingen, Germany) composed by a microchannel plate (MCP) high-rate intensifier (HRI), accepting gates down to 200 ps, at a rate between 20 and 80 MHz, a HRI delay generator (HDG), and a 640 × 480-pixel CCD camera, with 12-bit analog-to-digital (A/D) resolution, lens-coupled to the HRI. The camera is controlled by a dedicated PC computer.

The periodic patterns required by the optical sectioning technique (see next section) can be produced by reflection on a 606 × 654-pixel digital micromirror device module (DLP LightCrafter, Texas Instruments, Dallas, USA) or by transmission on a Ronchi ruling target.

**Figure 1:**
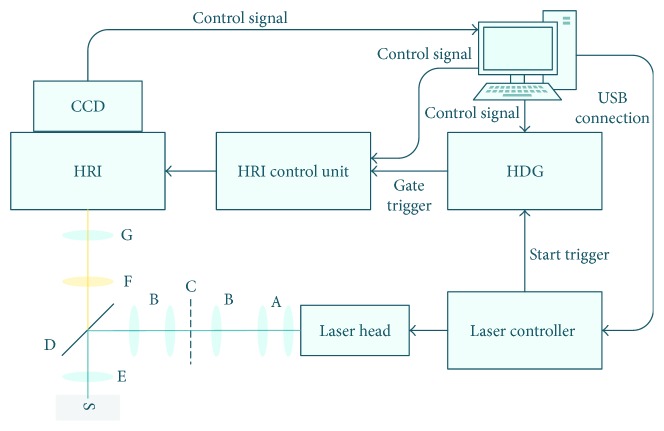
Time-gated fluorescence lifetime microscope. A: cylindrical lenses; B: positive lens; C: ruling target; D: dichroic mirror; E: infinity-corrected objective; F: fluorescence emission filter; G: infinity-corrected microscope module; S: sample.

The microscope optics were designed for use with infinity-corrected microscope objectives and were set up using individual optical components. Fluorescence discrimination is done through spectral filters: an excitation short-pass filter (Thorlabs FESH0450), a dichroic mirror (Chroma Q505lp), and an emission band-pass filter (Chroma HQ535/50m). The configuration of the microscope, when using a ruling target, is shown in Figure 1.

## 3. The HiLo Technique for Optical Sectioning

When imaging thick samples, time-gated FLIM is hampered by the effect of out-of-focus fluorescence that reduces the fluorescence lifetime contrast and results in incorrect fluorescence lifetime values [9]. On wide-field imaging techniques, this lack of optical sectioning can be addressed using frequency domain techniques, namely, by modulating the in-focus component of the image, followed by sideband demodulation and reconstruction. This is the basis of structured illumination microscopy, where a periodic pattern is projected at the focal plane of the microscope and the optically sectioned images are obtained by combining three or more structured images acquired for different phase shifts of the pattern [10]. It was already shown that structured illumination microscopy combined with FLIM provides accurate measurements of fluorescence lifetime at distinct overlapping layers [11].

Another spatial frequency domain optical sectioning technique is the so-called HiLo technique, introduced in 2008 by Lim et al. [12]. The HiLo technique has the advantage of using just two images, one obtained with structured illumination, implemented by a periodic or a speckle pattern, and another acquired under uniform illumination. In the structured image, the pattern is focused just through the depth of field of the microscope objective. Therefore, the in-focus contribution of the image is modulated and its low-frequency spectrum is shifted relatively to the frequency spectrum of the out-of-focus contribution and can be extracted by demodulation. The high frequencies of the in-focus contribution are extracted directly from the uniform image, using a high-pass filter, since this image does not contain any high-frequency component from the out-of-focus contribution. The final sectioned image is obtained by combining the low and high-frequency spectra of the in-focus contribution. This requires only an adjusting parameter to ensure a smooth transition between the two spectra [13].

It was shown that, using HiLo, FLIM measurements can be performed in the nanosecond range [14]. However, these authors reported a deviation for lower fluorescence lifetime values in the sectioned image (3.05 ± 0.35 ns against 3.20 ± 0.04 ns in the epi-fluorescence image of Alexa 488 *α*-tubulin immunostained HeLa cells), attributed to insufficient signal-to-noise ratio.

In this work, the HiLo algorithm was implemented in Matlab™. For reference purposes, we also used an ImageJ plugin available on the website of Jerome Mertz's group (http://biomicroscopy.bu.edu/resources/).

## 4. Instrument Response Function Deconvolution in RLD

As said, (1) is only accurate when the excitation laser pulse width is at least two orders of magnitude shorter than the measured fluorescence lifetime. In fact, the data *D*(*t*, *τ*) measured by a time-gated microscope corresponds to the convolution of the instrument response function (IRF) of the microscope, with the actual fluorescence decay illuminating the instrument's detector [15].

(2) $$\begin{array}{c} D\left(t,\tau \right)=IRF\left(t\right)*F\left(t,\tau \right), \end{array}$$

where *F*(*t*,*τ*) is the fluorescence decay, which for a single-exponential decay corresponds to

(3) $$\begin{array}{c} F\left(t,\tau \right)=N\left\{\left[H\left(t\right)+\frac{1}{\exp\left(T/\tau \right)}\right]\exp\left(-\frac{t}{\tau }\right)\right\}. \end{array}$$

Here, *H*(*t*) is the Heaviside step function or unit step function and *T* is the period of the train of excitation laser pulses. *N* is a parameter related to the total number of counts on the decay curve.

The microscope's IRF depends mainly on the width of the laser pulse and on the timing dispersion of the detector. The IRF can be measured using a reflector or a scatterer as sample.

The IRF affects the accuracy of measured fluorescence lifetime by overestimating its value. We verified this through computational simulation using as input 100 × 100 data arrays with constant fluorescence lifetime. From these arrays and using a time step of 1 ps, we obtained a volume of 25000 data arrays of 100 × 100 elements, where each element of the *m*th array is the value of the fluorescence decay curve for the *m*th time, calculated using (3).

Noise was then added to the data arrays. For the gated-intensified camera used in our system, noise can be modelled according to [16].

(4) $$\begin{array}{c} \sigma_{I}^{2}=k^{2}EN_{ph}+\sigma_{k}^{2}N_{ph}^{2}+\sigma_{CCD}^{2}, \end{array}$$

where *k* is the number of electrons in the CCD per detected photon, a value that depends on the image intensifier voltage, *E* is the excess noise factor, *N*_ph_ is the number of detected photons, *σ*_*k*_ is the standard deviation associated with fluctuations in the conversion constant *k*, and *σ*_CCD_ is the noise associated with CCD, like readout noise and dark current noise. For our simulations, we did not consider neither the thermal CCD noise nor the noise related to fluctuations in the *k* constant, which is significant only for very high MCP voltages, above those used in our instrument [16]. The *k* values (counts per electron) were obtained from measurements provided by the manufacturer of the gated-intensified camera, for different MCP voltages. The excess noise factor was calculated by interpolating the values presented by McGinty et al. for a similar camera [16]. The readout noise is 16 electrons per pixel.

**Figure 2:**
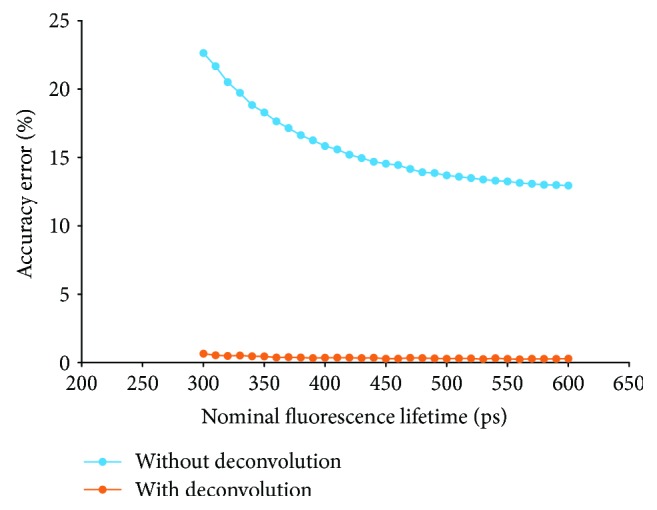
Accuracy error of the fluorescence lifetime calculated by the RLD technique without and with IRF deconvolution for different values of nominal fluorescence lifetime. The simulations were done considering a gate width of 1000 ps and a laser power level of 90%. The accuracy errors are presented as percentage of the nominal lifetimes.

Finally, the dataset was convoluted with the IRF. The fluorescence lifetime was determined using the RLD method. It is important to note that the windowing schemes have necessarily to be set up so that the beginning of the first integration window is close to the peak of the decay curve.  Figure 2 shows how the accuracy error of the RLD technique varies for different nominal fluorescence lifetimes, in the 300 to 600 ps range. The results presented were obtained using the IRF measured for a gate width of 1000 ps and a laser power of 90%. The accuracy error was calculated as the absolute deviation between the lifetime calculated by the RLD method and the nominal fluorescence lifetime used to generate the dataset, expressed as percentage of this nominal value.

We implemented an IRF deconvolution procedure to improve the accuracy of the RLD method. The algorithm starts by using the experimental images (*I*_0_exp__ and *I*_1_exp__) to calculate an array of lifetimes *τ*(*i*, *j*), using the RLD method. Then this array is used to generate a fluorescence decay dataset from (3). From this dataset, we select the two data arrays (*I*_0_sim__ and *I*_1_sim__) corresponding to the time positions of the *I*_0_exp__ and *I*_1_exp__ images and normalize them using the image *I*_0_exp__ as reference. The next step is to compute the square of the differences [(*I*_1_exp__ − *I*_1_sim__)^2^] that will be used as a measure of the goodness of the fit between experimental and simulated data. This procedure is repeated, while decreasing the elements of the array *τ*(*i*, *j*) until reaching the minimum value of the sum of the square of differences.

The performance of the IRF deconvolution algorithm was evaluated by computational simulation, using the datasets generated as explained in the previous paragraphs.  Figure 2 shows the accuracy error obtained after IRF deconvolution. The fluorescence lifetime values were obtained using the RLD method. The accuracy error was calculated as before.

## 5. Experimental Tests—Materials

The optical sectioning provided by the HiLo technique was evaluated using a glass target with a thin layer of a fluorescent dye. The target was mounted in a micrometer-controlled precision positioner and was axially scanned in 1 *μ*m steps through the focal plane of the microscope. For each axial step, we acquired an image and calculated its spatial frequency spectrum using a Fast Fourier Transform (FFT) algorithm. From each spectrum, we determined the value of the peak corresponding to the spatial frequency of the pattern projected onto the sample. This set of values corresponds to the axial point spread function of the microscope, and its FWHM is a measure of the optical sectioning. These measurements were performed with a ruling of 20 line pairs per millimeter and a Zeiss Achroplan 40x 0.75 W objective with a numerical aperture NA = 0.75.

To verify, on real fluorophores, the performance of the HiLo and RLD methods, we acquired FLIM images from samples with single-exponential fluorescence decay. We used two standard fluorophores: Erythrosin B and Coumarin 153. Both dyes were purchased in powder form from Sigma Aldrich (Darmstadt, Germany). Individual solutions of each dye in methanol (MeOH) were prepared. To obtain reference values, their fluorescence decays were measured with a time-correlated single-photon counting fluorescence lifetime microscope PicoQuant MicroTime 100 equipped with a pulsed diode laser equal to the one used in the time-gated microscope. The fluorescence decays were analyzed with a locally developed Microsoft Excel™ add-on software using the nonlinear least-squares fitting method with IRF deconvolution. The values obtained were 490.7 ps for the Erythrosin B and 3959.7 ps for the Coumarin 153.

The solutions were placed inside capillary tubes (100 *μ*m diameter) and imaged on the time-gated FLIM microscope. The microscope was operated with a gate width of 1000 ps, a gain of 5.2 counts per electron, and a laser power of 90%. Here, we used an Olympus Plan N 20x objective with a numerical aperture of 0.4, to have a larger field of view.

## 6. Results

### 6.1. Optical Sectioning with HiLo

**Figure 3:**
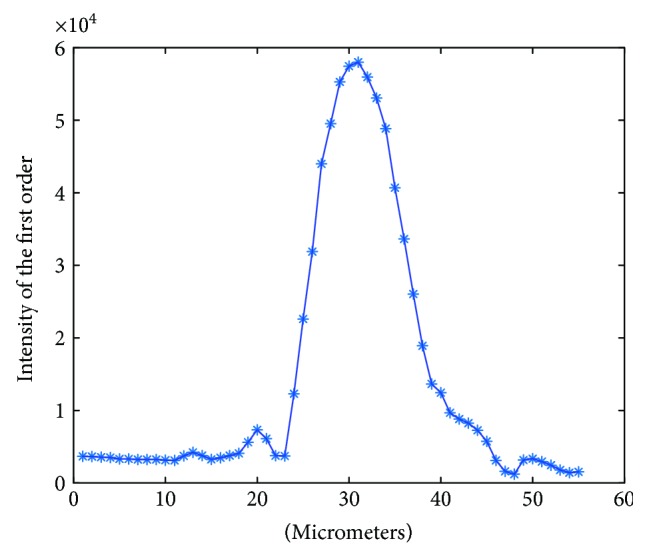
Axial PSF of the time-gated FLIM microscope. The FWHM is a measure of the microscope's optical sectioning.

Figure 3 shows the axial PSF of the time-gated FLIM microscope. By fitting a Gaussian function to the data, we measured a FWHM of 5.08 ± 0.40 *μ*m. This value is close to the theoretical optical sectioning value, which is equal to 4.84 *μ*m for the used objective.

**Figure 4:**
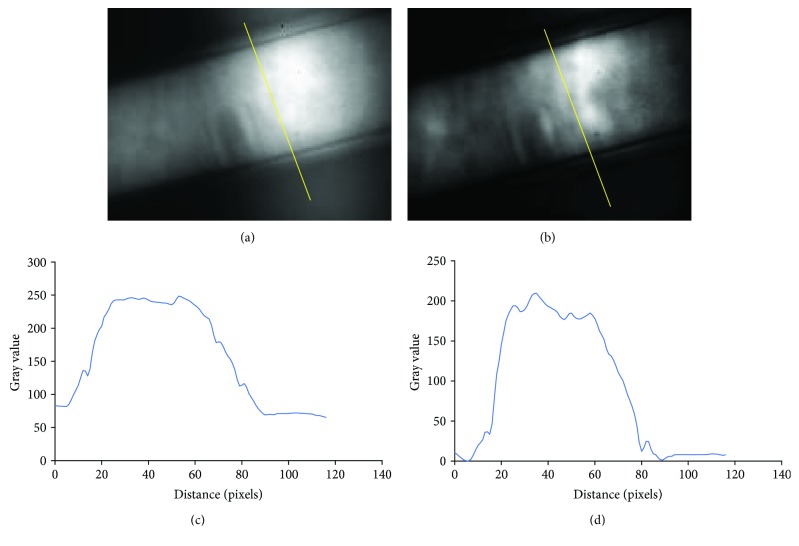
(a) Epi-fluorescence and (b) HiLo sectioned images of a stack of two capillary tubes filled with Coumarin 153 solution. (c, d) The intensity profiles obtained along the line superimposed on the corresponding image.

The effect of HiLo can be observed in Figure 4. Figures 4(a) and 4(c) show the wide-field epi-fluorescence image corresponding to two capillary tubes filled with Coumarin 153 solution, stacked perpendicularly to simulate a two-layer tissue, and the corresponding intensity profile for the line drawn on the image. Figures 4(b) and 4(d) show the equivalent data for the output of the HiLo algorithm. The comparison of the line profiles clearly shows the effectiveness of the sectioning technique.

Table 1 compares the fluorescence lifetimes measured using the RLD method between the epi-fluorescence images and their corresponding HiLo images. The measured values correspond to mean ± standard deviation of the intensity-weighted fluorescence lifetime image, with the weights of each pixel being the fractional contribution of the pixel to the total fluorescence intensity. Intensity weighting is used to suppress the noisy fluorescence lifetime values. It is possible to verify that the HiLo method does not affect significantly the mean value of the measured fluorescence lifetime. However, in the case of the solution with the longer fluorescence lifetime, there is a large degradation of the measurement precision.

### 6.2. IRF Deconvolution

Table 1 also shows that the measured fluorescence lifetimes do not coincide with the reference values. This deviation, which is very large for the shorter lifetime of the Erythrosine solution, results from the finite width of the time-gated FLIM microscope IRF and should be corrected by the deconvolution process.

**Figure 5:**
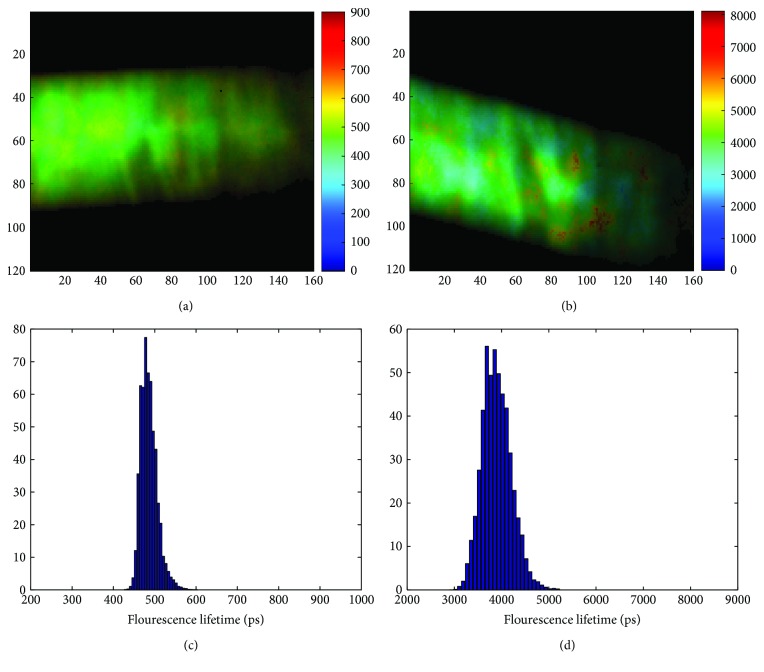
Fluorescence lifetime images (a, b) and histograms (c, d) for HiLo processed images of capillary tubes filled with Erythrosine B (a, c) and Coumarin 153 (b, d) solutions, after running the IRF deconvolution algorithm. By comparing the histograms with the values presented in Table 1, it is possible to verify that the IRF deconvolution algorithm improves the accuracy of the fluorescence lifetimes measured with RLD.

Figure 5 shows the intensity-weighted fluorescence lifetime images and histograms for HiLo processed images of capillary tubes filled with Coumarin 153 and Erythrosine B solutions, after running the IRF deconvolution algorithm. The IRF deconvolution improved greatly the accuracy of the lifetime of the Erythrosine B solution. The lifetime after deconvolution was 491.43 ± 30.2 ps. The algorithm was also able to improve slightly the accuracy of the Coumarin 153 lifetime, obtaining a lifetime of 3999.8 ± 452.1 ps. Measured values correspond to mean ± standard deviation of the intensity-weighted fluorescence lifetime image.

## 7. Conclusions

We evaluated the application of the HiLo method for optical sectioning on a wide-field time-gated FLIM microscope based on a picosecond pulsed diode laser. The HiLo method could produce optical sectioning on thick (200 *μ*m) samples without degrading the accuracy of the measured fluorescence lifetimes, in the subnanosecond and nanosecond ranges. However, it was observed a significant degradation in the precision of the longer lifetime. This is due to the small intensity variation between the two images used by the RLD algorithm, resulting from the longer fluorescence lifetime. In our experiments, we used contiguous windowing gates. We verified that we could increase the precision of the fluorescence lifetime by increasing the separation between the two gates. This is an example of the limitations of the RLD method: the need of a priori knowledge on the expected lifetimes to maximize the signal-to-noise ratio and, consequently, the precision of the measurements.

The measurements confirmed the great impact of the microscope's IRF in the accuracy of shorter lifetimes. The IRF deconvolution could be successfully applied to the HiLo image and improved greatly the accuracy of the Erythrosine B lifetime, resulting in a measured value very close to the reference value. This result opens the possibility of using instruments based on low-cost pulsed diode laser sources and on the RLD method to determine accurately fluorescence lifetimes in the subnanosecond range. This is a relevant result when imaging living tissues, on applications where offline processing of the lifetime images is allowed.

## Figures and Tables

**Table 1:** Fluorescence lifetimes measured for Coumarin 153 and Erythrosine B solutions in capillary tubes using the RLD technique on epi-fluorescence and HiLo images. The measured values correspond to mean ± standard deviation of the intensity-weighted fluorescence lifetime image. The reference lifetimes were obtained with a time-correlated single-photon counting microscope.

Solution	Reference lifetime (ps)	Epi-fluorescence image lifetime (ps)	HiLo image lifetime (ps)
Coumarin 153	3959.7	3825.6 ± 198.1	3846.6 ± 463.5
Erythrosine B	490.7	604.2 ± 40.4	613.2 ± 42.0
